# Dominant negative effects by inactive Spa47 mutants inhibit T3SS function and *Shigella* virulence

**DOI:** 10.1371/journal.pone.0228227

**Published:** 2020-01-24

**Authors:** Jamie L. Burgess, Heather B. Case, R. Alan Burgess, Nicholas E. Dickenson

**Affiliations:** Department of Chemistry and Biochemistry, Utah State University, Logan, Utah, United States of America; Centre National de la Recherche Scientifique, Aix-Marseille Université, FRANCE

## Abstract

Type three secretion systems (T3SS) are complex nano-machines that evolved to inject bacterial effector proteins directly into the cytoplasm of eukaryotic cells. Many high-priority human pathogens rely on one or more T3SSs to cause disease and evade host immune responses, underscoring the need to better understand the mechanisms through which T3SSs function and their role(s) in supporting pathogen virulence. We recently identified the *Shigella* protein Spa47 as an oligomerization-activated T3SS ATPase that fuels the T3SS and supports overall *Shigella* virulence. Here, we provide both *in vitro* and *in vivo* characterization of Spa47 oligomerization and activation in the presence and absence of engineered ATPase-inactive Spa47 mutants. The findings describe mechanistic details of Spa47-catalyzed ATP hydrolysis and uncover critical distinctions between oligomerization mechanisms capable of supporting ATP hydrolysis *in vitro* and those that support T3SS function *in vivo*. Concentration-dependent ATPase kinetics and experiments combining wild-type and engineered ATPase inactive Spa47 mutants found that monomeric Spa47 species isolated from recombinant preparations exhibit low-level ATPase activity by forming short-lived oligomers with active site contributions from at least two protomers. In contrast, isolated Spa47 oligomers exhibit enhanced ATP hydrolysis rates that likely result from multiple preformed active sites within the oligomeric complex, as is predicted to occur within the context of the type three secretion system injectisome. High-resolution fluorescence microscopy, T3SS activity, and virulence phenotype analyses of *Shigella* strains co-expressing wild-type Spa47 and the ATPase inactive Spa47 mutants demonstrate that the N-terminus of Spa47, not ATPase activity, is responsible for incorporation into the injectisome where the mutant strains exhibit a dominant negative effect on T3SS function and *Shigella* virulence. Together, the findings presented here help to close a significant gap in our understanding of how T3SS ATPases are activated and define restraints with respect to how ATP hydrolysis is ultimately coupled to T3SS function *in vivo*.

## Introduction

Type three secretion systems (T3SS) are highly conserved complex multi-component nano-machines that inject proteins from the cytoplasm of pathogenic bacteria into eukaryotic host cells [1–4]. Once injected, the bacterial effector proteins subvert host cell function to support infection and evade immune responses [5, 6]. Many important bacterial pathogens such as *Yersinia*, *Salmonella*, *Pseudomonas*, *Burkholderia*, *Escherichia*, and *Shigella* rely on one or more T3SSs as critical virulence factors necessary to cause disease [3, 7–9]. While the injected T3SS effector proteins are specific to each pathogen’s infection mechanism and replicative niche, they are all secreted through a highly-conserved syringe and needle-like injectisome [10, 11]. The injectisome consists of a basal body that spans the periplasmic space and anchors the apparatus to the bacterial inner and outer membranes, a hollow needle that extends from the basal body beyond the lipopolysaccharide layer, and a protein tip complex that serves as an environmental sensor and provides access to the host cell cytoplasm by penetrating the host membrane [10–13]. In addition, a multi-protein cytosolic sorting platform is located just below the apparatus basal body and is believed to play important roles in the recognition and unfolding of effector proteins prior to secretion [14–16]. The thermodynamic driving force(s) that supports formation of the apparatus and protein secretion through the needle, however, remain largely unclear and somewhat controversial. The flagellar T3SS has recently been shown to utilize proton and sodium electrochemical gradients to drive a flow of H^+^ and Na^+^ ions through a transmembrane channel within the basal body of the injectisome to fuel a putative proton/protein antiporter mechanism [17, 18], however, efficient protein secretion additionally relies on ATP hydrolysis by an associated T3SS ATPase located at the base of the apparatus [19–21]. Like much of the actions supporting and regulating type three secretion, the specific contributions of the associated T3SS ATPase are not well understood. It is clear, however, that most, if not all, T3SSs contain a highly conserved ATPase that is most active in oligomeric form and resides just below the apparatus export gate at the base of the T3SS [22–26]. Dissecting the specific mechanisms through which T3SS ATPases function and precisely how they facilitate protein secretion has proven exceptionally challenging due to long-standing difficulties expressing and purifying soluble/active forms of many of the isozymes as well as accessing and interrogating both the dormant monomeric and activated oligomeric forms of the enzymes.

Here, we take advantage of the ability to express and purify stable recombinant monomeric and oligomeric stoichiometries of the *Shigella* T3SS ATPase Spa47 to define the role of complex dynamics in enzyme activation and efficiency. Additionally, co-expression experiments in which *Shigella* produce both wild-type and engineered ATPase inactive Spa47 point mutants uncover a dominant negative effect on *Shigella* T3SS formation, effector secretion, and overall *Shigella* virulence phenotype. The implications of these findings together with high-resolution live-cell fluorescence microscopy localizing each of the Spa47 constructs within individual *Shigella* bacteria are discussed with respect to contributions of Spa47-catalyzed ATP hydrolysis in T3SS function and pathogen virulence.

## Materials and methods

### Materials

Wild-type *S*. *flexneri* corresponds to the serotype 2a 2457T strain originally isolated in 1954 [27]. The *S*. *flexneri* Spa47 null strain was engineered by Abdelmounaaïm Allaoui as described by Jouihri et al [28]. Rabbit polyclonal antibodies against IpaC and IpaD and the pWPsf4 expression plasmid were generous gifts from William Picking and Wendy Picking (University of Kansas). *E*. *coli* strains and 2x ligation mix were from Novagen (Madison, WI). Restriction enzymes, the pTYB21 protein expression plasmid, polymerase chain reaction (PCR) buffer, Phusion High-Fidelity polymerase, and chitin resin were purchased from New England Biolabs (Ipswich, MA). Oligonucleotide primers and the synthesized *spa47* gene were from Integrated DNA Technologies (Coralville, IA). The N-terminal GFP pWPsf4 vector was designed in house and synthesized by General Biosystems (Morrisville, NC). The Superdex 16/600 size exclusion and 5-ml Q FF columns were purchased from General Electric (Pittsburgh, PA). ATP was from Sigma-Aldrich, and α-^32^P-ATP was from PerkinElmer Life Sciences. Dithiothreitol (DTT) and ampicillin were from Gold Biotechnology (St. Louis, MO). Defibrinated sheep red blood cells were from Colorado Serum Company (Denver, CO). All other solutions and chemicals were of reagent grade.

### Cloning

The *spa47* gene was previously purchased as a double-stranded gBlock product from Integrated DNA Technologies with modifications for cloning into the expression plasmid pTYB21 as described previously [22]. Briefly, the *spa47* gene was digested using SapI and PstI restriction enzymes and then ligated into the expression plasmid pTYB21, which encodes an N-terminal chitin-binding domain and intein linker. The ligated product was transformed into *E*. *coli* Nova Blue cells by heat shock and sequence verified by Sanger sequencing (Genewiz, Inc., South Plainfield, NJ). The *spa47* gene was additionally cloned into the plasmid pWPsf4 for constitutive expression in *Shigella* by introducing NdeI and BamHI restriction sites at the 5′ and 3′ ends, respectively, and ligating into the digested vector backbone. Sequences were again verified by Sanger sequencing prior to transformation into an electro-competent *S*. *flexneri* Spa47 null strain via electroporation. The Spa47 point mutations used in this study and the Spa47^Δ1–79^ N-terminal truncation construct were generated in both pTYB21 and pWPsf4 using inverse PCR followed by sequence verification and transformation into *E*. *coli* and *S*. *flexneri*, respectively. The N-terminal GFP-Spa47 chimeras were generated by cloning the wild-type and mutant *spa47* genes into the NdeI/BamHI digested N-terminal GFP pWPsf4 backbone engineered for this study. The constructs were then transformed into a Nova Blue *E*. *coli* cloning strain, sequence verified, and electroporated into the *S*. *flexneri spa47* null strain.

### Protein expression and purification

Each of the Spa47 constructs encoded in pTYB21 were transformed into *E*. *coli* Tuner (DE3) cells and were expressed and purified as previously described [22, 29, 30]. Briefly, the expression strains were grown to an OD_600_ of approximately 0.8 in Terrific Broth medium containing 0.1 mg/ml ampicillin at 37 °C and 200 RPM. The culture was then cooled to 17 °C before induction with 1 mM isopropyl β-D-1-thiogalactopyranoside (IPTG) for ∼20 hours (17 °C, 200 rpm). All subsequent steps were carried out at 4 °C unless otherwise stated. The cells were pelleted by centrifugation, resuspended in binding buffer (20 mM Tris, 500 mM NaCl, pH 7.9) containing 0.2 mM 4-(2-aminoethyl) benzenesulfonyl fluoride hydrochloride (AEBSF) and lysed by sonication. The clarified supernatant was exposed to a chitin affinity column to capture the chitin-binding domain-intein-Spa47 chimera and purified Spa47 was eluted by on column cleavage of the intein domain in binding buffer containing 50 mM freshly-added DTT. The elution fractions were pooled and diluted to a final buffer composition of 20 mM Tris, 100 mM NaCl, 10 mM DTT, pH 7.9 prior to negative selection over a Q Sepharose FF anion exchange column. The purified Spa47 in the flow through was concentrated using an ultra centrifugal filter unit with a 30-kDa molecular mass cutoff and further purified/characterized using a Superdex 200 16/600 size exclusion column equilibrated with 20 mM Tris, 100 mM NaCl, 5 mM DTT, pH 7.9. As validated previously [22], Spa47 concentrations were determined using in-gel densitometry with Coomassie staining and BSA as a standard. All concentrations are reported in monomer concentration units for consistency and clarity.

### Spa47 ATP hydrolysis assay

A multiple time point activity assay was used to determine the effect of protein concentration on ATP hydrolysis rates for monomeric and oligomeric Spa47. The assay was carried out under conditions we have optimized and described previously [31, 32]. Briefly, the reactions were initiated by combining increasing concentrations of either monomeric or oligomeric Spa47 with a prepared ATP solution, resulting in a final concentration of 10 mM MgCl_2_, 1 mM total ATP, and 0.5 μCi (∼300 nM) α-^32^P-ATP. Samples were rapidly quenched at desired time points with a final concentration of 250 mM ethylenediaminetetraacetic acid (EDTA). The level of ATP hydrolysis was quantified by separating α-^32^P-ATP substrate from the α-^32^P-ADP product using thin layer chromatography (TLC). The TLC plates were exposed to a storage phosphor screen, the separated species were detected with a Storm PhosphorImager (Molecular Dynamics), and the product concentrations were quantified using the associated ImageQuant software (Molecular Dynamics). The same multiple time point activity assay was used to determine the effect of combining Spa47 active site mutants with wild-type Spa47. Wild-type monomeric Spa47 was mixed with each tested Spa47 mutant to produce a final concentration of 0.45 μM wild-type monomeric Spa47 and 16.2 μM monomeric Spa47 mutant.

### *S*. *flexneri* invasion of epithelial cells

Invasion phenotype of a *S*. *flexneri* strain lacking the gene for Spa47, a strain expressing wild-type Spa47, and strains containing a polycistronic Spa47/Spa47 mutant expression system were determined by a gentamicin protection assay. The details of the gentamycin protection assay are described elsewhere [33]. Briefly, the polycistronic expression system used in this study included the gene for wild-type Spa47 in the first cloning site of the plasmid and the gene for either wild-type Spa47 or a Spa47 mutant in the second cloning site. The genes in both sites are under transcriptional control of the same promoter and include identical, but independent, ribosome binding sites upstream of each gene, resulting in constitutive expression of both proteins from a single RNA transcript. Sterile 24-well plates were seeded with passaged HeLa cells and grown overnight in DMEM supplemented with 10% fetal calf serum, penicillin, and streptomycin at 100% relative humidity, 37 °C, and 5% CO_2_. The *S*. *flexneri* strains were streaked onto tryptic soy agar plates containing 0.025% Congo red and grown overnight at 37 °C. Small cultures containing appropriate antibiotics were inoculated from the agar plates and grown to OD_600_ of ∼0.5 at 37 °C and 200 rpm. Equivalent bacterial loads were introduced to the cultured HeLa cells, and the plates were centrifuged at 1000 × g for 5 min to synchronize contact between the bacteria and HeLa cells. The inoculated cells were incubated at 37 °C for 30 min, rinsed to remove extracellular bacteria, and treated with 50 μg/ml gentamicin to selectively kill any remaining *Shigella* that had not successfully invaded the HeLa cells. The HeLa cells were then lysed with 1% agarose in water and overlaid with a LB agar solution. Overnight incubation at 37 °C resulted in *Shigella* colony formation from the previously internalized bacteria, allowing a quantitative comparison of invasion phenotype among the tested *Shigella* strains.

### *Shigella*-induced erythrocyte hemolysis

The effects of the engineered polycistronic *spa47* constructs on T3SS-mediated hemolysis of red blood cells were determined using a slightly modified version of a previously described red blood cell hemolysis assay [34]. Briefly, Spa47 null *S*. *flexneri* expressing the Spa47 construct combinations described above were grown overnight on TSA-Congo red plates and a small number of isolated colonies were used to inoculate 10 ml of tryptic soy broth containing appropriate antibiotics. The cultures were grown to an OD_600_ of approximately 0.5, collected by centrifugation, and gently resuspended in 200 μl of PBS. Fifty microliters of each bacterial mix were then combined with ∼5 × 10^8^ defibrinated sheep red blood cells in a 96-well microtiter plate and centrifuged at 2300 x g, 25 °C for 10 min to initiate contact between the bacteria and the red blood cells. The mixture was then incubated at 37 °C for 1 h and each condition resuspended following the addition of 100 μl of PBS. The resuspended mix was centrifuged at 2300 x g and 4 °C for 10 min to separate the bacteria and insoluble red blood cell components from the hemoglobin that was released as a result of red blood cell lysis by *Shigella*. The levels of released hemoglobin in the supernatant were quantified by measuring absorbance at 545 nm and compared with the levels resulting from a Spa47 null *S*. *flexneri* strain and a strain expressing wild-type Spa47.

### Quantitation of *S*. *flexneri* T3SS translocator secretion

The small diazo dye Congo red effectively induces active secretion of translocator proteins through the *Shigella* T3SS by mimicking the natural trigger resulting from host cell membrane interaction [35]. In this study, the polycistronic expression system described above was used to simultaneously express wild-type Spa47 and an ATPase inactive Spa47 mutant within a Spa47 knockout *Shigella* strain. The protocol for the Congo red assay has been described in detail elsewhere [36]. Briefly, a *S*. *flexneri* strain lacking the gene for Spa47, a strain expressing wild-type Spa47, and strains expressing the engineered polycistronic wild-type Spa47 and Spa47 mutants were grown overnight on TSA-Congo red plates, and a small number of isolated colonies were used to inoculate 10 ml of tryptic soy broth containing appropriate antibiotics. Cultures were grown at 37 °C to an OD_600_ of approximately 1.0 before they were cooled on ice to temporarily slow protein expression and secretion. The cultures were centrifuged and rinsed to separate the bacteria from the culture supernatant and any proteins that had been secreted up to this point. The cells were then resuspended in pH 7.4 sodium phosphate buffer containing 0.28 mg/ml Congo red and were incubated at 37 °C for 1 hour to promote active T3SS protein secretion. Cultures were then chilled on ice for 5 min to limit further secretion and the bacteria were separated from the protein-containing supernatant by centrifugation at 13,000 × g for 15 min at 4 °C. The secreted proteins within the supernatant from each strain were separated using SDS-PAGE, transferred to PVDF membranes by western blot, and probed using anti-IpaC rabbit polyclonal antibodies and fluorescent Alexa 647 conjugated goat anti-rabbit secondary antibodies. Secreted IpaC levels were detected and compared using a Bio-Rad ChemiDoc imaging system and the associated Image Lab analysis software. As validated previously [37], a monoclonal antibody against the cytoplasmic enzyme glyceraldehyde-3-phosphate dehydrogenase (GAPDH) was used as a cytoplasmic control in the western blot to ensure that the IpaC detected in the supernatant was secreted through the injectisome and was not the result of cell lysis. A complete set of whole cell extract GAPDH controls is included in S3 Fig.

### *Shigella* T3SS injectisome detection by immuno-fluorescence based flow cytometry

The ability of *S*. *flexneri* strains used in this study to assemble extracellular portions of the injectisome was investigated using flow cytometry coupled with fluorescence detection. As described previously [21], each of the tested *S*. *flexneri* strains were streaked onto TSA-Congo red plates, and a small number of isolated colonies were used to inoculate 15 ml of tryptic soy broth containing appropriate antibiotics. The cultures were grown to an OD_600_ of approximately 0.8 at 37 °C and 200 RPM, collected by centrifugation at 4 °C, and gently rinsed with PBS. The cells were then chemically fixed for 15 min in 4% formaldehyde in PBS at room temperature (20–22 °C). The fixed cells were rinsed and labeled with rabbit polyclonal antibodies against the needle tip protein IpaD and then treated with an Alexa 647 conjugated goat anti-rabbit secondary antibody to fluorescently label the cells for detection by flow cytometry. The cells were rinsed again with PBS and analyzed using a BD Accuri C6 flow cytometer collecting 100,000 instances/run. The resulting data sets were analyzed with De Novo FCS Express 5 flow cytometry software to determine the extent of T3SS injectisome assembly in each of the tested strains.

### Live-cell TIRFM detection and localization of fluorescent Spa47 constructs

*S*. *flexneri* expressing N-terminal GFP Spa47 chimeras were grown to an OD_600_ of ~1.0. Ten milliliters of each culture were centrifuged at 2272 x g for 4 minutes to gently pellet the bacteria. The bacteria were washed once with room temperature PBS and then resuspended in 500 μL of PBS and diluted as necessary to achieve the desired density of bacteria in the microscopy images. Twenty microliters of the diluted bacteria were placed between two No. 1.5 cover slides and fluorescence images were collected using an Olympus IX51 total internal reflection fluorescence microscope (TIRF-M) (Olympus, Center Valley, PA) equipped with a 100x TIRF objective (1.49 NA, infinity corrected, achromat). The 488 nm and 640 nm laser lines of a Toptica Chrome laser system (Toptica Photonics, Farmington, NY) were directed through the objective and the sample fluorescence collected in epifluorescence geometry and filtered through appropriate dichroic mirrors and long pass filters prior to collection on a Hamamatsu ORCA-Flash4.0 CMOS camera (Hamamatsu, Hamamatsu City, Japan). Excitation parameters and image collection were controlled with cellSense Dimension software (Olympus, Center Valley, PA).

## Results and discussion

### Monomeric Spa47 is converted to the activated homo-oligomer *in vitro*

**Fig 1:**
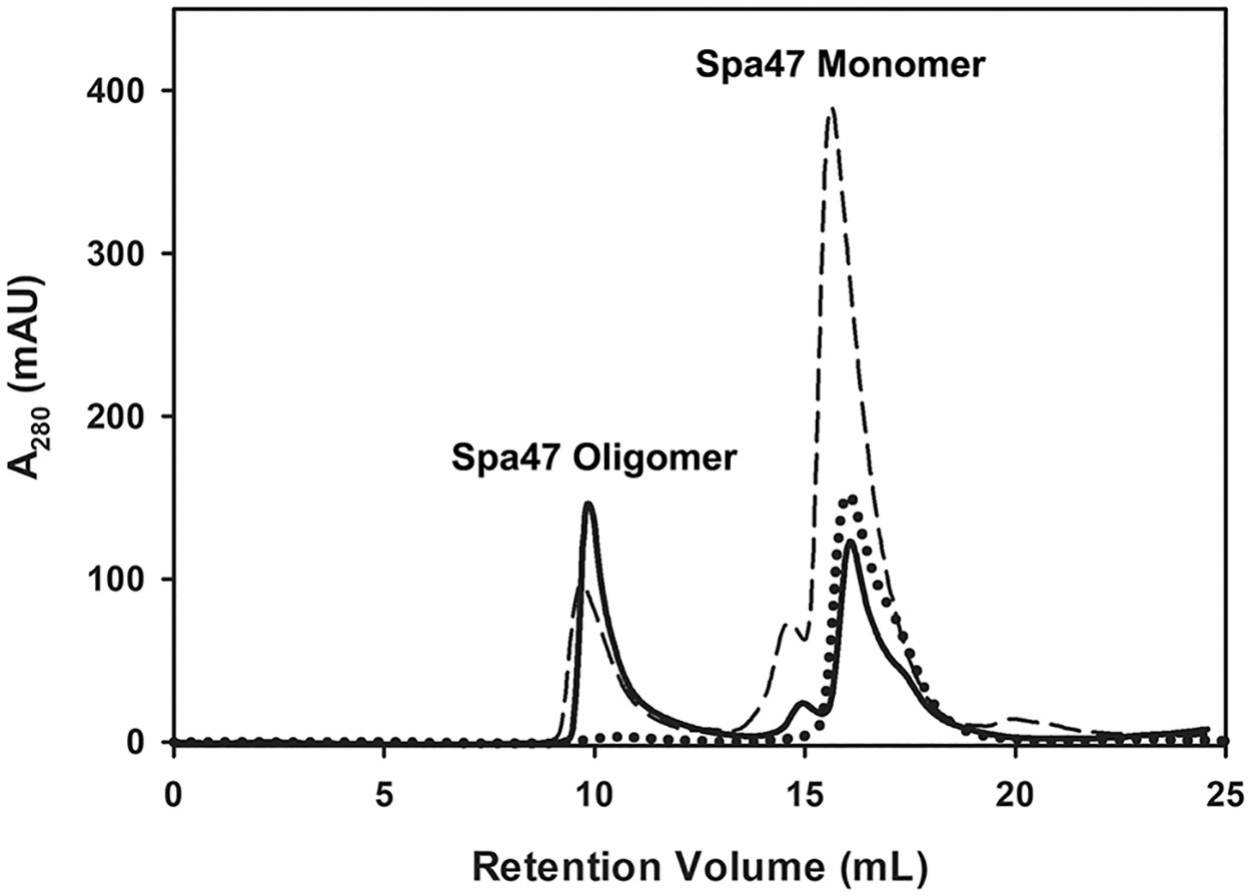
Isolated monomeric Spa47 is converted *in vitro* to its activated homo-oligomeric form at high protein concentrations. Purification of recombinant Spa47 results in distinct monomeric and homo-oligomeric species that can be isolated using a Superdex200 10/300 size exclusion column (long dashed line). Isolation and re-evaluation of the monomeric species results in a nearly exclusively monomeric distribution (dotted line). Concentration of the isolated monomeric species to ~200 μM transformed much of the monomeric population to an oligomeric species that elutes identically to the previously characterized Spa47 homo-oligomer (solid line).

Spa47 purifies *in vitro* as stable monomeric and oligomeric species, with the oligomer displaying significantly higher ATPase activity than the monomer [21, 22, 30]. Recent insights into Spa47 activation suggest that oligomerization supports ATP hydrolysis by contributing amino acid sidechains from adjacent Spa47 protomers to form a single complete active site [21], supporting the observed enhancement of *in vitro* ATPase activity exhibited by oligomeric *Shigella* Spa47 and several related T3SS ATPases [22, 24, 25, 38–40]. Spa47 has served and continues to serve as a valuable model for studying T3SS ATPase activation and regulation as monomeric and oligomeric Spa47 are easily isolated via size exclusion chromatography (SEC) and isolated Spa47 fractions maintain their original stoichiometry for several weeks when stored at the concentrations collected directly from the sizing column (~1–20 μM). Interestingly, concentrating monomeric Spa47 to ~200 μM prior to re-evaluating oligomeric state via SEC resulted in the formation of stable Spa47 oligomers that elute identically to the activated oligomeric Spa47 species that is isolated following the original expression and purification process (Fig 1). Additionally, this *in vitro* conversion of Spa47 monomer to oligomer resulted in an increase in ATPase activity from 0.11 ± 0.01 (μmol ADP/min) /mg Spa47 to 1.50 ± 0.03 (μmol ADP/min) /mg Spa47 (S1 Fig), providing the first example of *in vitro* formation of stable activated Spa47 oligomers and demonstrating that monomeric Spa47 is fully capable of converting to the activated oligomeric species. These results identify a means to generate active Spa47 oligomers from recombinantly expressed and purified Spa47 monomers and lends support to the hypothesis that controlling Spa47 oligomer state provides a potent mechanism for regulating *Shigella* virulence *in vivo*.

### Monomeric Spa47 exhibits concentration-dependent ATPase activity

**Fig 2:**
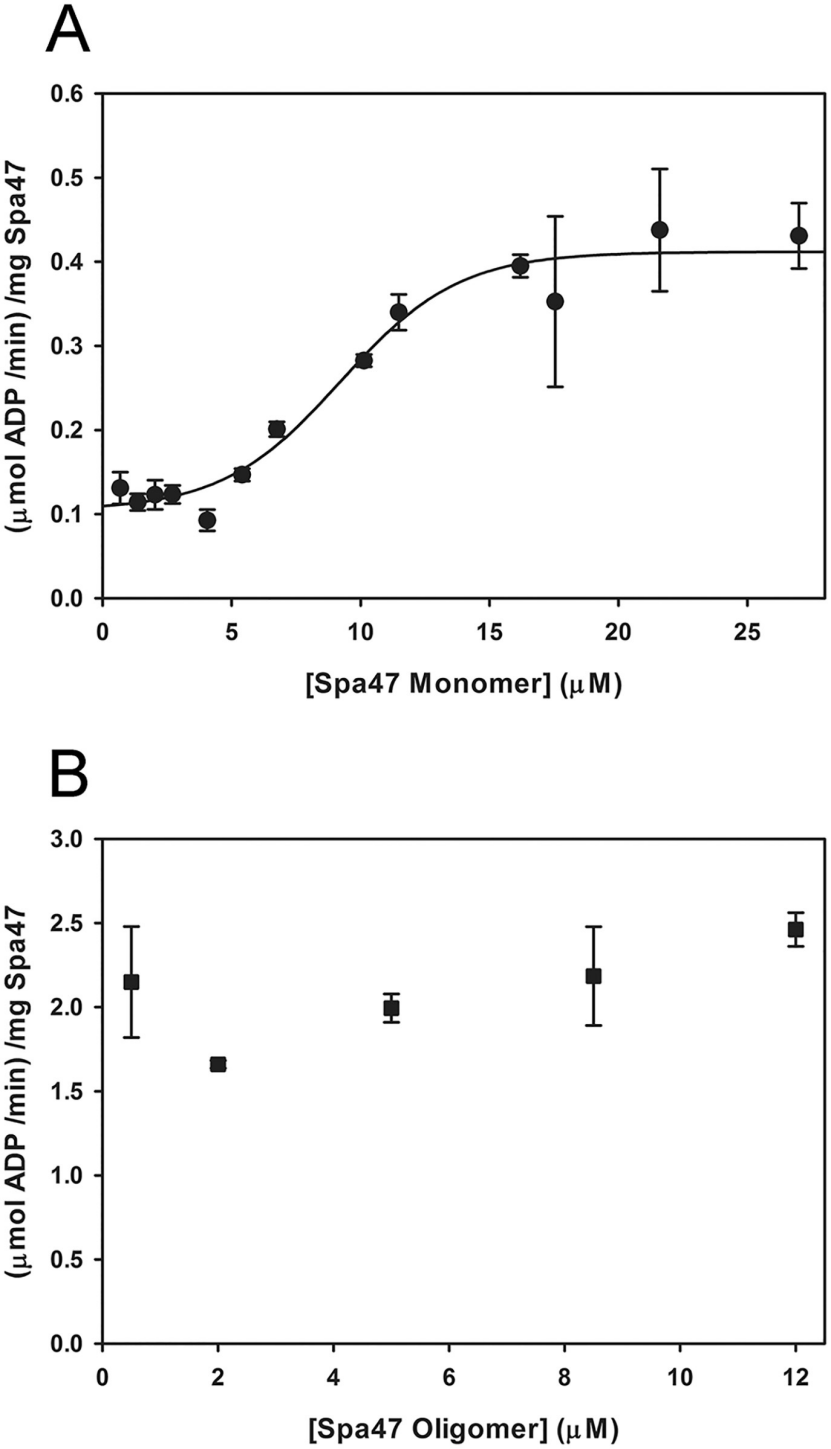
Monomeric Spa47 exhibits concentration dependent ATPase activity while oligomeric Spa47 activity is concentration independent. A) Radiolabeled ATP hydrolysis assays show that increasing concentrations of monomeric Spa47 increase enzyme efficiency. B) The ATPase activity of oligomeric Spa47 is not dependent on enzyme concentration. These data are representative of triplicate measurements spanning two independent protein preparations.

The high protein concentration required to convert Spa47 monomers to stable oligomers *in vitro* is clearly far beyond the protein concentrations achieved directly from the size exclusion column when isolating monomeric and oligomeric Spa47 following expression and initial purification. However, even at concentrations much lower than required to convert monomeric Spa47 to stable active oligomer, the monomeric Spa47 provides minimal, yet robust ATPase activity that we hypothesize results from transient Spa47 interactions in solution that provide the necessary contributions from at least two Spa47 monomers to complete the enzyme active site and support ATP hydrolysis. We tested this hypothesis by quantifying the ATP hydrolysis rates (normalized for enzyme concentration) of both monomeric and oligomeric Spa47 species as a function of enzyme concentration (Fig 2). It is important to note that the upper protein concentration limit for the ATPase activity dilution series for both species was determined by the concentrations that could be achieved directly from the size exclusion column. As a result, the protein was not concentrated following elution from the sizing column, ensuring the stoichiometry of the Spa47 used in the assay. The monomeric Spa47 ATPase activity exhibited a strong positive sigmoidal response to enzyme concentration with an overall 3-fold increase in activity level, consistent with formation of concentration-dependent transient complexes consisting of the ATP substrate and at least two molecules of Spa47 (Fig 2A). In contrast, the oligomeric Spa47 species activity was insensitive to enzyme concentration (Fig 2B), representing the optimally active species achieved in this assay.

### Monomeric ATPase inactive Spa47 point mutants form transient active hetero-oligomeric complexes with monomeric wild-type Spa47

**Fig 3:**
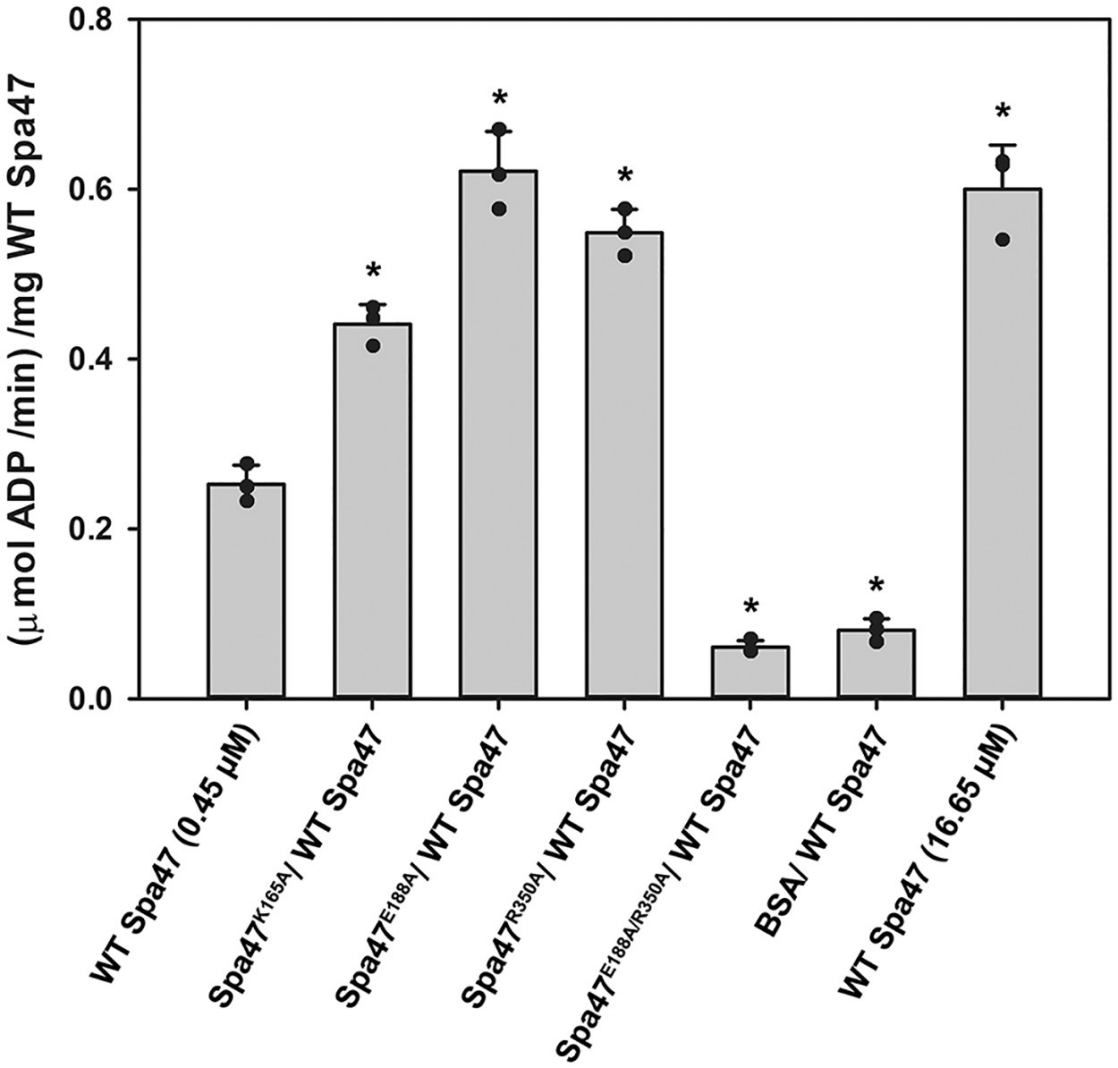
The addition of ATPase inactive Spa47 mutants influences wild-type monomeric Spa47 ATPase activity. Excess of each ATPase inactive monomeric Spa47 active site mutant used in this study (16.2 μM) was added to wild-type monomeric Spa47 (0.45 μM) prior to quantifying ATP hydrolysis by the Spa47 mixture. The presented data represent the mean ± standard deviation of triplicate measurements. *Indicates a significant difference in activity level relative to 0.45 μM wild-type Spa47 monomer (one-way ANOVA followed by a Dunnett’s post test, p ≤ 0.05)).

The positive cooperativity observed for monomeric wild-type Spa47 monomer (Fig 2A) was attributed to the concentration-dependent formation of transient oligomeric species that provide complete ATPase active sites at the junction of at least two Spa47 protomers. To interrogate the nature of *in vitro* oligomer formation by monomeric Spa47, a series of mixing experiments were performed in which monomeric isolates of previously characterized [21, 22], ATPase inactive Spa47 point mutants were introduced to a solution of monomeric wild-type Spa47. The addition of Spa47^K165A^, Spa47^E188A^, and Spa47^R350A^ unexpectedly each enhanced the ATPase activity of wild-type Spa47 to levels similar to that observed for an equivalent concentration (16.65 μM) of wild-type Spa47 (Fig 3). Realizing, however, that the active site is specifically comprised of K165 and E188 from a single face of one protomer and R350 from the opposite face of an adjacent protomer, each of the inactive point mutants still contain one face that can contribute to the complementary half of the active site in wild-type Spa47. In other words, the K165A and E188A mutants both maintain a functional R350 that can contribute to the K165/E188 face of the wild-type monomer and likewise the R350A mutant can contribute K165 and E188 to the R350 face of the wild-type Spa47 monomer, thus increasing the effective concentration of complete/functional active sites with the addition of independently inactive Spa47 mutants. This was further confirmed by the addition of an inactive Spa47 mutant carrying both E188A and R350A mutations, resulting in a marked decrease in activity that is nearly identical to the decrease that is observed following the addition of BSA to WT monomeric Spa47. As expected, none of the ATPase inactive mutants had any effect on the activity of oligomeric wild-type Spa47, consistent with a lack of protomer exchange within the stable oligomeric complex (S2 Fig).

### *C*o-expression of wild-type and inactive Spa47 point mutants results in dominant negative *Shigella* virulence phenotypes

**Fig 4:**
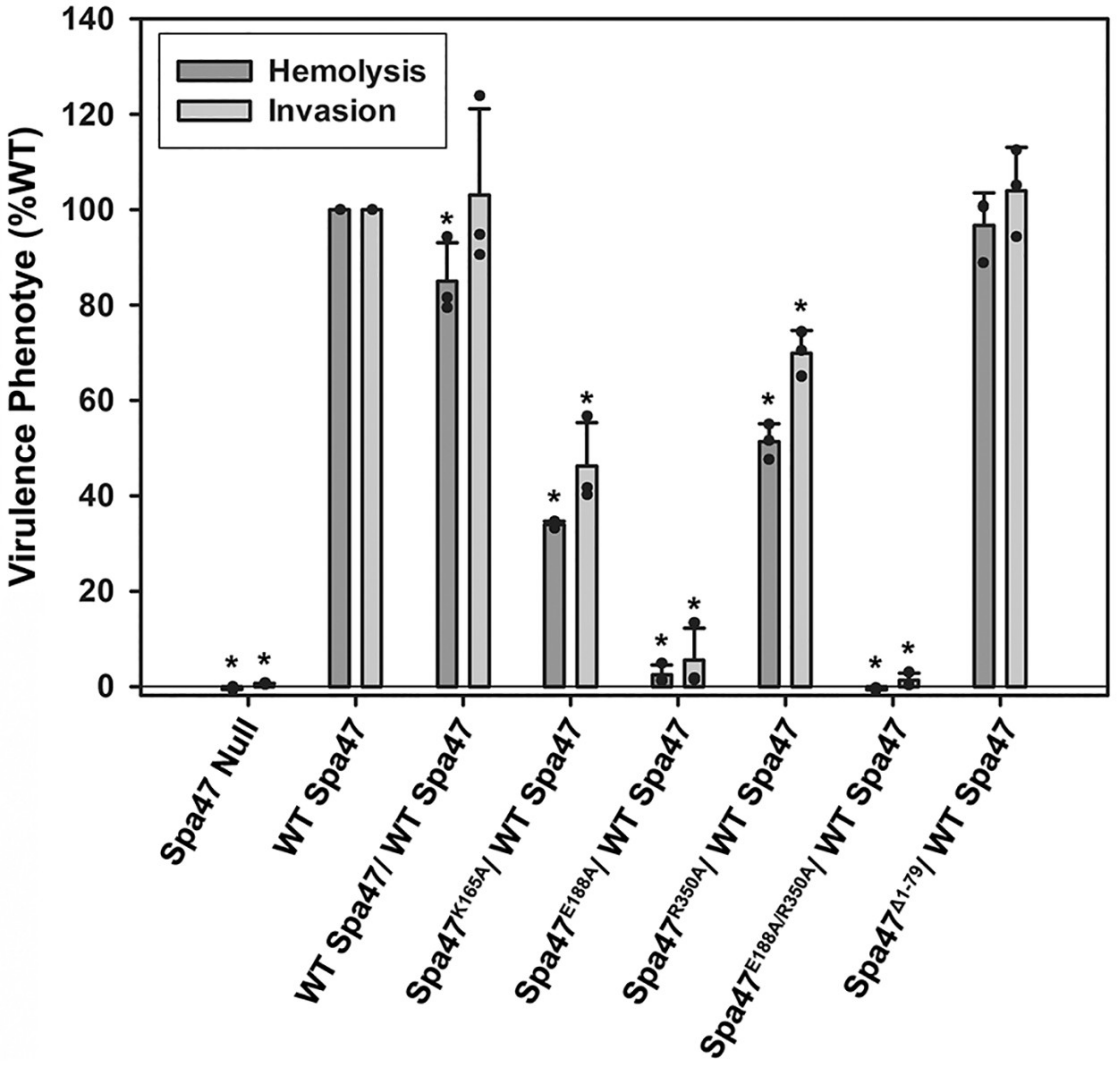
Expression of inactive Spa47 mutants within a wild-type *Shigella* background produces dominant negative effects on pathogen virulence. The negative impact of expressing inactive Spa47 mutants in the presence of wild-type Spa47 is shown using red blood cell hemolysis and cellular invasion assays (relative to a *Shigella* strain constitutively expressing wild-type Spa47 from a single gene). The data represent the mean ± standard deviation of three independent experiments spanning at least two biological replicates. *Indicates a significant difference in phenotype relative to the wild-type Spa47 strain (one-way ANOVA followed by a Dunnett’s post test, p ≤ 0.05)).

Each of the inactive Spa47 point mutants described in this study were independently co-expressed in *Shigella* with wild-type Spa47 to determine the impact the presence of the mutants has on an otherwise wild-type Spa47 and T3SS. The effect of the co-expression on *Shigella* virulence was measured by monitoring the mutant *Shigella* strains’ ability to both lyse red blood cells and invade cultured eukaryotic host cells. The hemolysis and invasion results in Fig 4 clearly show that a Spa47 null *Shigella* strain is unable to either lyse red blood cells or invade eukaryotic cells; the result of a previously described loss in injectisome formation and T3SS protein secretion [21]. Complementing the Spa47 null *Shigella* strain with either a single gene for wild-type Spa47 or using a polycistronic expression vector encoding wild-type Spa47 from both of the two cloning sites restores function of the T3SS and supports both red blood cell hemolysis and cellular invasion (Fig 4). On the other hand, polycistronic co-expression of wild-type Spa47 with the active site point mutants Spa47^K165A^, Spa47^E188A^, and Spa47^R350A^ results in a significant reduction in both hemolysis and invasion phenotype. This is in contrast with the *in vitro* results in Fig 3 showing that the addition of these mutants to wild-type Spa47 increased ATPase activity through the formation of additional active sites in the complexes, suggesting that while interaction between a wild-type Spa47 and inactive Spa47 point mutant can facilitate ATP hydrolysis, it does not support proper function *in vivo*. Furthermore, co-expression of wild-type Spa47 with the inactive Spa47 double mutant, Spa47^E188A/R350A^, nearly eliminates *Shigella* virulence while co-expression of wild-type Spa47 with the oligomerization deficient and ATPase inactive Spa47^Δ1–79^ N-terminal truncation mutant has no effect on either hemolysis or invasion. Together, these findings suggest that contributions from Spa47 beyond just ATP hydrolysis are necessary to support *Shigella* virulence and that the mutant lacking the N-terminal domain is likely unable to interact with and interfere in the role of wild-type Spa47 *in vivo*. This is consistent with recent findings in which the related T3SS ATPase, EscN, was found to form a donut-shaped homo-hexamer with the “central stalk” homolog, EscO, threaded through the central pore of the structure [41]. In this geometry, the inclusion of a protomer that is unable to fully contribute to the active site on either face of the adjacent protomer would ultimately inhibit the rotary ATP hydrolysis mechanism and prevent coupling of ATP hydrolysis to T3SS function, preventing T3SS function and pathogen virulence (as observed in this study).

### Co-expression of wild-type and inactive Spa47 point mutants inhibits *Shigella* T3SS effector protein secretion and T3SS apparatus formation

**Fig 5:**
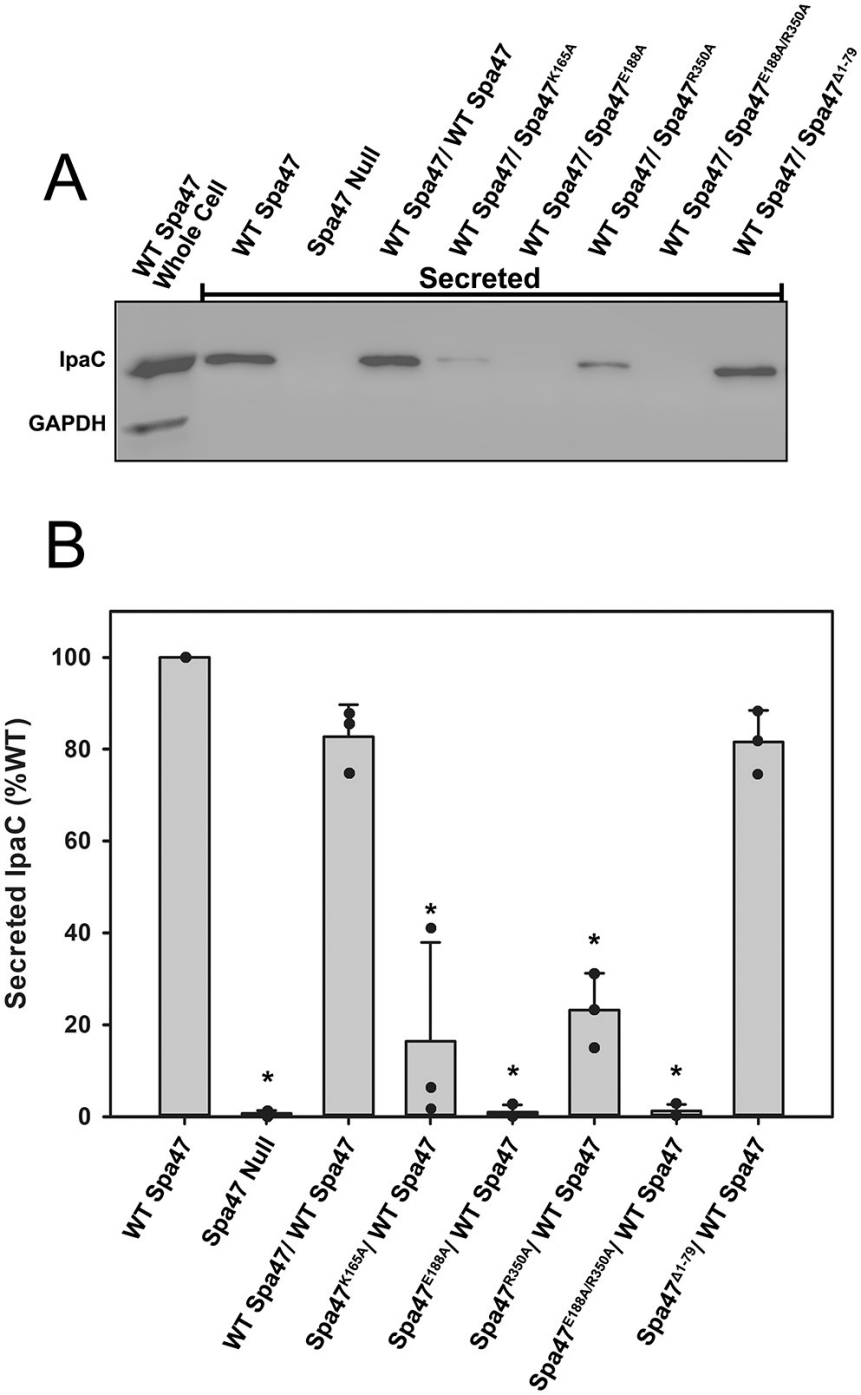
T3SS effector protein secretion is reduced in *Shigella* strains co-expressing wild-type and inactive Spa47. A) Representative western blot of the secreted T3SS effector protein IpaC following Congo red activation of *Shigella* cultures expressing the indicated combinations of Spa47. The cytoplasmic enzyme GAPDH served as a control and was observed in the whole cell extracts (WCE) but not in the supernatant containing the secreted IpaC protein. B) The Western blot intensities were quantified and plotted as the mean ± standard deviation from three blots using independent biological replicates. The quantified protein levels are displayed as a percentage relative to those secreted by the *Shigella* strain expressing wild-type Spa47. *Indicates a significant difference in IpaC secretion level relative to the wild-type Spa47 complement strain (one-way ANOVA followed by a Dunnett’s post test, p ≤ 0.05)).

**Fig 6:**
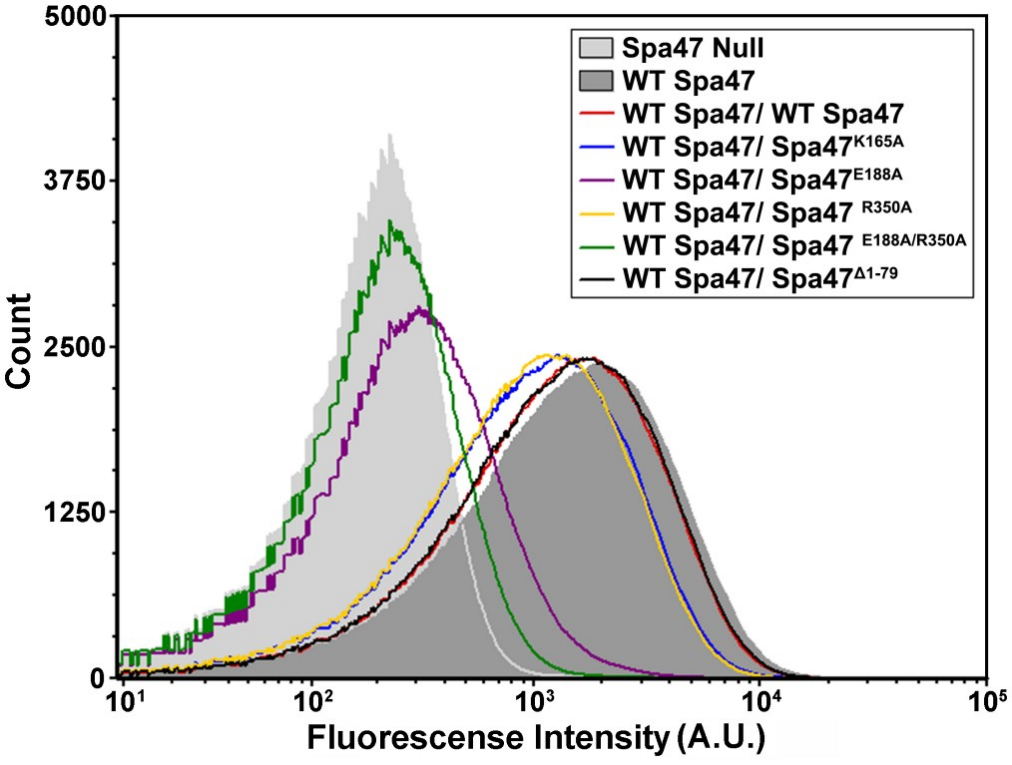
Flow cytometry fluorescence intensity histograms comparing extracellular T3SS injectisome tip protein presentation by *Shigella* strains expressing combinations of wild-type and mutant Spa47 constructs. The tested *Shigella* strains were each treated with primary rabbit polyclonal antibodies against the T3SS tip protein IpaD and Alexa Fluor 647 conjugated goat anti-rabbit secondary antibodies to fluorescently label T3SS proteins on the bacterial surface. The histograms include 100,000 individual intensity measurements per condition and are representative of two independent biological replicates.

We have shown previously that eliminating expression of Spa47 entirely or attempting to complement a Spa47 null strain with one of the ATPase inactive Spa47 mutants used in this study renders the mutant *Shigella* strain avirulent as it is unable to secrete T3SS effector proteins or properly form the external portion of the T3SS apparatus [21]. Expecting that the reduction in *Shigella* virulence phenotype illustrated in Fig 4 may be the result of similar deficiencies, the effect of co-expressing the ATPase inactive Spa47 mutants with wild-type Spa47 was examined with respect to both *Shigella* T3SS effector secretion and apparatus formation. As described previously [35], the small diazo dye Congo red was used to activate *Shigella* T3SS protein secretion in each of the strains used in this study. Consistent with the phenotype results in Fig 4, a significant decrease in secretion levels of the effector protein IpaC is observed in the strains co-expressing wild-type Spa47 with any of the inactive Spa47 point mutants, while the strains expressing wild-type Spa47 from a single gene, expressing wild-type Spa47 from two genes within the polycistronic expression vector, and co-expressing wild-type Spa47 with the Spa47^Δ1–79^ oligomerization deficient N-terminal truncation construct each maintain wild-type secretion levels (Fig 5). Furthermore, T3SS apparatus formation was tested for each of the *Shigella* strains in this study by fluorescently immuno-labeling the apparatus tip protein IpaD and evaluating its presence on the bacterial surface using flow cytometry. IpaD provides a sensitive means of evaluating external apparatus formation as multiple copies constitutively reside at the tip of the injectisome needle and its location at the tip of the ~45 nm needle is highly accessible to the antibody probes. Fig 6 includes fluorescence histograms comprised of fluorescence intensity measurements from 100,000 individual *Shigella* per condition. As we have reported previously [21, 28], the Spa47 null *Shigella* strain is unable to form an external T3SS needle and results in a fluorescence intensity histogram indistinguishable from background while complementing the null strain with wild-type Spa47 reinstates its ability to form a proper injectisome and shifts the histogram to higher intensity values. Not surprisingly, based on their Congo red secretion profiles, the strains co-expressing wild-type Spa47 and either the Spa47^E188A^ or the Spa47^E188A/R350A^ mutants are severely handicapped with respect to forming type three secretion apparatus. The *Shigella* strain co-expressing wild-type Spa47 and Spa47^Δ1–79^ and the strain expressing wild-type Spa47 from two genes within the polycistronic expression vector, however, each result in fluorescence intensity histograms that appear essentially identical to the wild-type Spa47 complemented *Shigella* strain, suggesting that Spa47 activity and T3SS apparatus formation remain unaffected in these mutants. Interestingly, the *Shigella* strains co-expressing wild-type Spa47 with either Spa47^K165A^ or Spa47^R350A^ display a minimal reduction in apparatus formation (Fig 6) that corresponds to intermediate levels of effector secretion (Fig 5) and modest, yet statistically significant, reductions in hemolysis and invasion phenotypes (Fig 4), suggesting that perhaps these mutants are not efficiently incorporated into the active Spa47 complex or that interactions within the apparatus mitigate the negative effect(s) that result from these single mutations.

### The N-terminal domain of Spa47, not ATPase activity, is responsible for Spa47 localization to the *Shigella* T3SS injectisome

**Fig 7:**
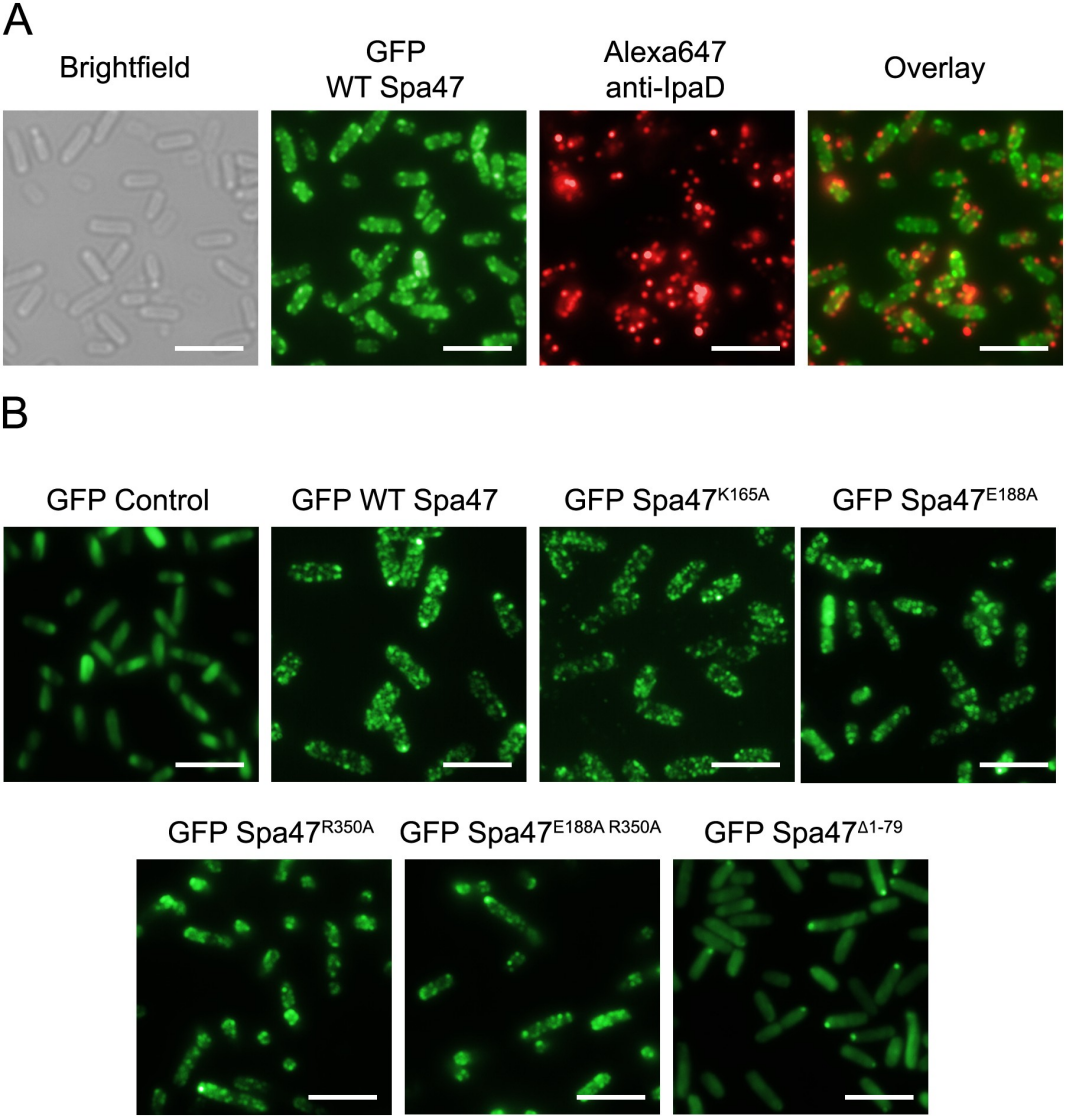
Total internal reflection fluorescence microscopy (TIRFM) localizes wild-type and mutant Spa47 constructs to the *Shigella* T3SS. A) Immunofluorescent labeling of the injectisome needle tip protein IpaD results in punctate fluorescence that corresponds to individual T3SS injectisome needle tips (red) and co-localizes with punctate signal from cytoplasmic GFP-tagged wild-type Spa47 (green). B) Expression of the ATPase inactive GFP-Spa47 point mutants used in this study also results in punctate fluorescence distribution indicative of incorporation of the Spa47 chimeras into the injectisome sorting platform. Both the GFP control and the Spa47^Δ1–79^ construct appear predominantly diffuse throughout the cytoplasm. Scale bars = 5 μM.

The phenotype results from this study are consistent with a mechanism where the addition of ATPase inactive Spa47 to a wild-type *Shigella* background results in a dominant negative effect by preventing efficient coupling of ATP hydrolysis by native wild-type Spa47 to protein secretion through the apparatus, reducing overall *Shigella* virulence. It is difficult to predict the precise origin of the observed dominant negative effect, though the established link between Spa47 ATPase activity and *Shigella* virulence phenotype suggest that the inactive Spa47 point mutants are either preventing ATP hydrolysis *in vivo* by the co-expressed wild-type Spa47 or that they are preventing the proper coupling of ATP hydrolysis to T3SS function. Some insight into this question came during the preparation of this manuscript as the Strynadka group solved a 3.3Å Cryo-EM structure of the homo-hexameric *E*. *coli* T3SS ATPase, EscN, in complex with the central stalk protein, EscO [41]. The high-resolution structure provides strong evidence for a rotary ATPase mechanism similar to that of F_1_F_o_ ATP synthase [41, 42]. While no other high-resolution homo-oligomeric T3SS ATPase structures have been solved to date, it is widely believed that most, if not all, T3SS-expressing bacteria incorporate a homo-hexamer of their respective T3SS ATPase at the base of the T3SS [14, 41, 43]. If Spa47 relies on a rotary-type ATPase mechanism similar to that of F- and V-type ATPases, then incorporation of even a single ATPase inactive protomer into the complex would prevent ATPase activity altogether. To determine whether each of the mutants in this study are capable of incorporating into the injectisome, we developed fluorescent N-terminal GFP-Spa47 chimeras of wild-type Spa47 and each of the Spa47 mutants used in this study. Importantly, incorporating the N-terminal GFP tag does not hinder Spa47 function *in vivo* as the fluorescent chimera complements cellular invasion of a Spa47 null *Shigella* strain to 113 ± 15% of wild-type (S1 Table). The fluorescent GFP-Spa47 constructs were then expressed in *Shigella* to visualize the localization of each construct within the bacteria using live-cell total internal reflection fluorescence microscopy (TIRFM). In this scenario, TIRF provides a much higher fluorescence signal-to-noise ratio than standard epi fluorescence microscopy due to specific excitation of fluorophores located within approximately 100 nm of the interface between the glass coverslip and aqueous buffer solution, eliminating out of focus fluorescence and clearly identifying punctate fluorescence if the GFP-Spa47 chimeras are localized to the T3SS injectisome. Fig 7A demonstrates the fluorescently immuno-labeled IpaD located at the tips of several injectisome needle assemblies localizes to GFP-tagged wild-type Spa47 located at the bacterial membrane. Fig 7B shows that expression of GFP alone into an otherwise native *Shigella* strain results in diffuse fluorescence and no evidence of specific localization. Interestingly, co-expressing the GFP-Spa47 mutant chimeras with non-tagged wild-type Spa47 found that each of the independently inactive Spa47 mutants also result in punctate fluorescent signal consistent with forming oligomers at the membrane and localization to T3SS sorting platforms. The GFP-Spa47^Δ1–79^ chimera, however, does not incorporate into the T3SS sorting platform at the base of the injectisome and results in a generally diffuse fluorescence signal with the construct accumulating at a single pole of several bacteria within the field of view. Polar accumulation is commonly observed for protein aggregates and a similar polar accumulation has been observed for the *Salmonella* sorting platform protein SpaO in mutants lacking critical T3SS proteins [44], suggesting that without proper interactions with additional injectisome components, the T3SS ATPases may engage in aberrant interactions and localize to the bacterial pole. In the case of Spa47, it is interesting to note that the Spa47^Δ1–79^ construct which is unable to properly localize within *Shigella* is not only oligomerization-deficient [21], but is also unable to interact with its putative molecular chaperone, MxiN [30]. Taken together, the TIRFM localization studies show that wild-type Spa47 and each of the ATPase-inactive Spa47 point mutant-GFP chimeras are incorporated into the T3SS injectisome while Spa47^Δ1–79^ is not. This suggests that the dominant negative phenotypes observed in the presence of the inactive Spa47 point mutants is a result of disruption of Spa47 function within the apparatus and that oligomerization and/or interaction with MxiN likely drives Spa47 incorporation into the maturing injectisome independently of ATP hydrolysis by the incorporated enzyme. This is consistent with studies in the *Salmonella* SPI-1 and flagellar T3SSs showing that the MxiN homologs OrgB and FliH are critical components of the injectisome sorting platform that also facilitate T3SS ATPase localization to the C-ring of the injectisome basal body.[44, 45]

## Conclusions

Taken together, the data from this study uncover valuable mechanistic details describing the oligomerization-dependent activation of the *Shigella* T3SS ATPase Spa47 with strong conservation among T3SSs and T3SS ATPases suggesting that the findings will also provide a foundation for expanding this work to related pathogens. Moreover, we show that Spa47 provides a potentially unique benefit in that it purifies as stable monomeric and nucleotide-independent oligomeric species with the monomer exhibiting concentration-dependent basal level ATPase activity that results from the formation of transient oligomers with interfacial active sites formed between two Spa47 protomers. The significantly higher ATPase activity of the Spa47 homo-oligomers, however, is not concentration dependent and results from stable long-lived interactions that provide a primed active site at each protomer interface within the oligomer, suggesting that the oligomeric form represents the highest stoichiometry species achieved *in vitro* and a valuable tool for studying T3SS ATPase activation. Live cell fluorescence imaging of Spa47 and a series of engineered Spa47 mutants show that the N-terminal domain, not ATPase activity of the individual Spa47 constructs, is responsible for localization of Spa47 to the injectisome and suggest that incorporation of the ATPase-inactive Spa47 mutants into the apparatus is responsible for the dominant negative effect observed in T3SS function and *Shigella* virulence. Clearly, additional studies are needed to untangle the specific role(s) that T3SS ATPases play in T3SS function, but these findings emphasize the importance of Spa47 oligomerization in both enzyme activation and T3SS function and support the F_1_/V_1_-like rotary hydrolysis mechanism that has been proposed to fuel protein secretion through the T3SS-needle complex.

## Supporting information

S1 Fig Oligomerization of isolated monomeric Spa47 enhances ATPase activity. As shown in Fig 1, isolated monomeric Spa47 is converted to stable oligomers following concentration to >200 μM. Kinetic evaluation of the newly formed oligomeric species shows that the oligomeric Spa47 is significantly more active than the original monomeric species.
(TIF)

S2 Fig ATPase activity of isolated wild-type Spa47 oligomers is unaffected by the addition of ATPase inactive Spa47 mutants. Excess of each ATPase inactive oligomeric Spa47 active site mutant used in this study (1.8 μM) was added to wild-type oligomeric Spa47 (0.45 μM) prior to quantifying ATP hydrolysis by the Spa47 mixture. The presented data represent the mean ± standard deviation of triplicate measurements. The ATPase activity of the wild-type Spa47 oligomer was unaffected by the addition of the ATPase inactive Spa47 mutants (one-way ANOVA, p ≤ 0.05), suggesting that the oligomers are stable and do not undergo protomer exchange *in vitro*.
(TIF)

S3 Fig Congo red induced whole cell extract controls. Congo red induction of T3SS effector secretion was performed and the secretion profiles shown in Fig 5. Following isolation of the *Shigella* culture supernatant, the bacterial cells were lysed and probed via SDS-PAGE/Western blot analysis for cytoplasmic levels of the effector protein IpaC (red) and the cytoplasmic control glyceraldehyde-3-phosphate dehydrogenase (GAPDH, green).
(TIF)

S1 Table Cellular invasion phenotype of *Shigella* expressing an engineered N-terminal GFP Spa47 chimera. ^a^The ability of each tested *Shigella* strain to invade cultured host cells was measured by a standard gentamicin protection assay. Invasion results are presented as the percent invasion by the *S*. *flexneri* strain expressing wild-type Spa47. Experiments were performed in triplicate and results represent the mean ± standard deviation of three independent biological replicates.
(DOCX)

S2 Table Strains and plasmids used in this study. (DOCX)

## Abbreviations

AEBSF: 4-(2-aminoethyl) benzenesulfonyl fluoride
BSA: bovine serum albumin
DMEM: Dulbecco’s modified eagle medium
DTT: dithiothreitol
EDTA: ethylenediaminetetraacetic acid
GADPH: glyceraldehyde-3-phosphate dehydrogenase
GFP: green fluorescent protein
IPTG: isopropyl β-D-1-thiogalactopyranoside
LB: Luria broth
PBS: phosphate buffered saline
PVDF: polyvinylidene difluoride
SEC: size exclusion chromatography
T3SS: type three secretion system
TIRF: total internal reflection fluorescence
TIRFM: total internal reflection fluorescence microscopy
TLC: thin layer chromatography
TSA: tryptic soy agar
WCE: whole cell extract
