# Supplementary figures and images for: Dominant negative effects by inactive Spa47 mutants inhibit T3SS function and Shigella virulence
Source: PLoS One. 2020 Jan 24;15(1):e0228227. doi: 10.1371/journal.pone.0228227 (PMC6980540; doi:10.1371/journal.pone.0228227)

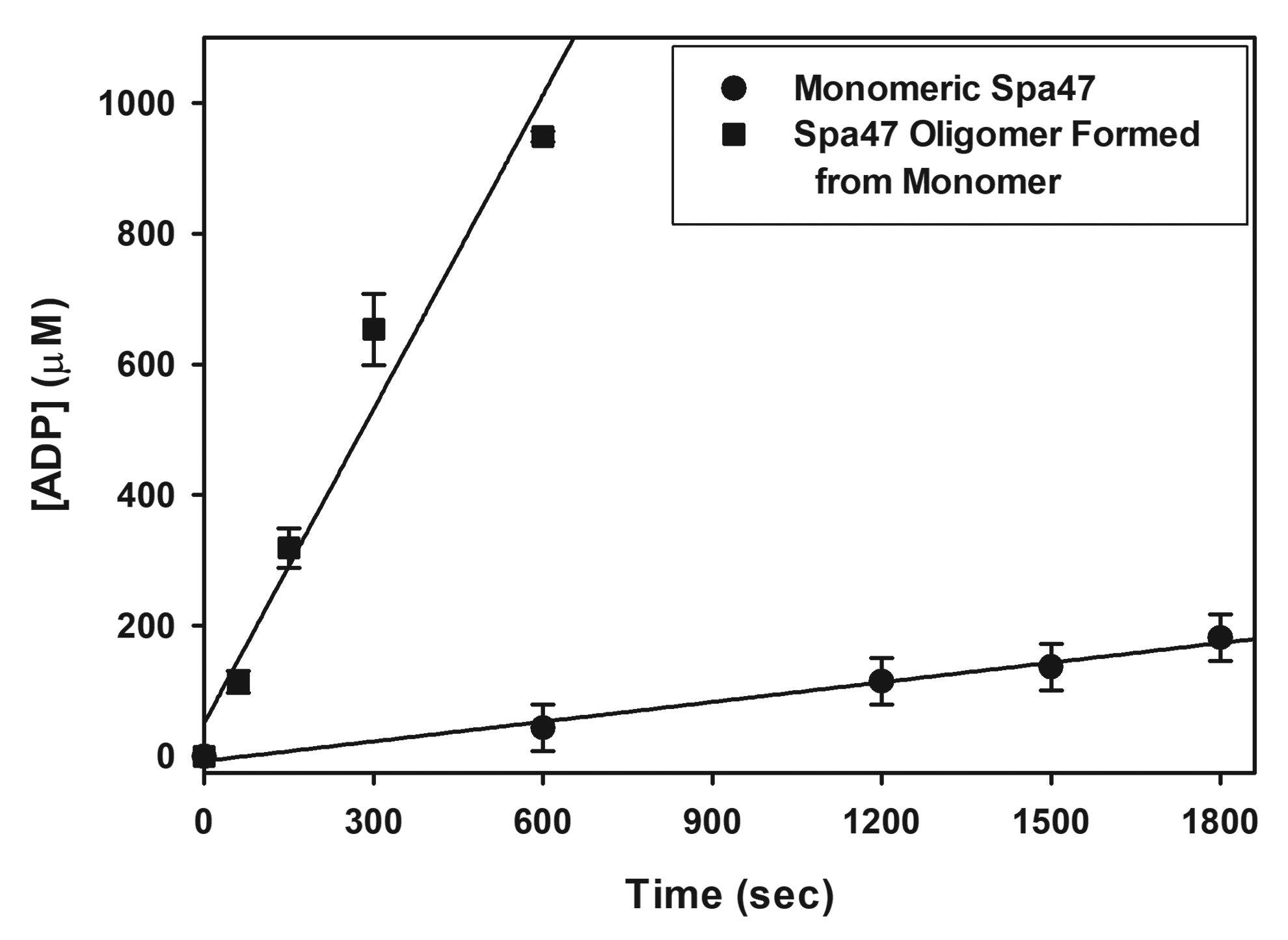

Supplement: S1 Fig — As shown in Fig 1, isolated monomeric Spa47 is converted to stable oligomers following concentration to >200 μM. Kinetic evaluation of the newly formed oligomeric species shows that the oligomeric Spa47 is significantly more active than the original monomeric species. (TIF) [file pone.0228227.s001.tif]

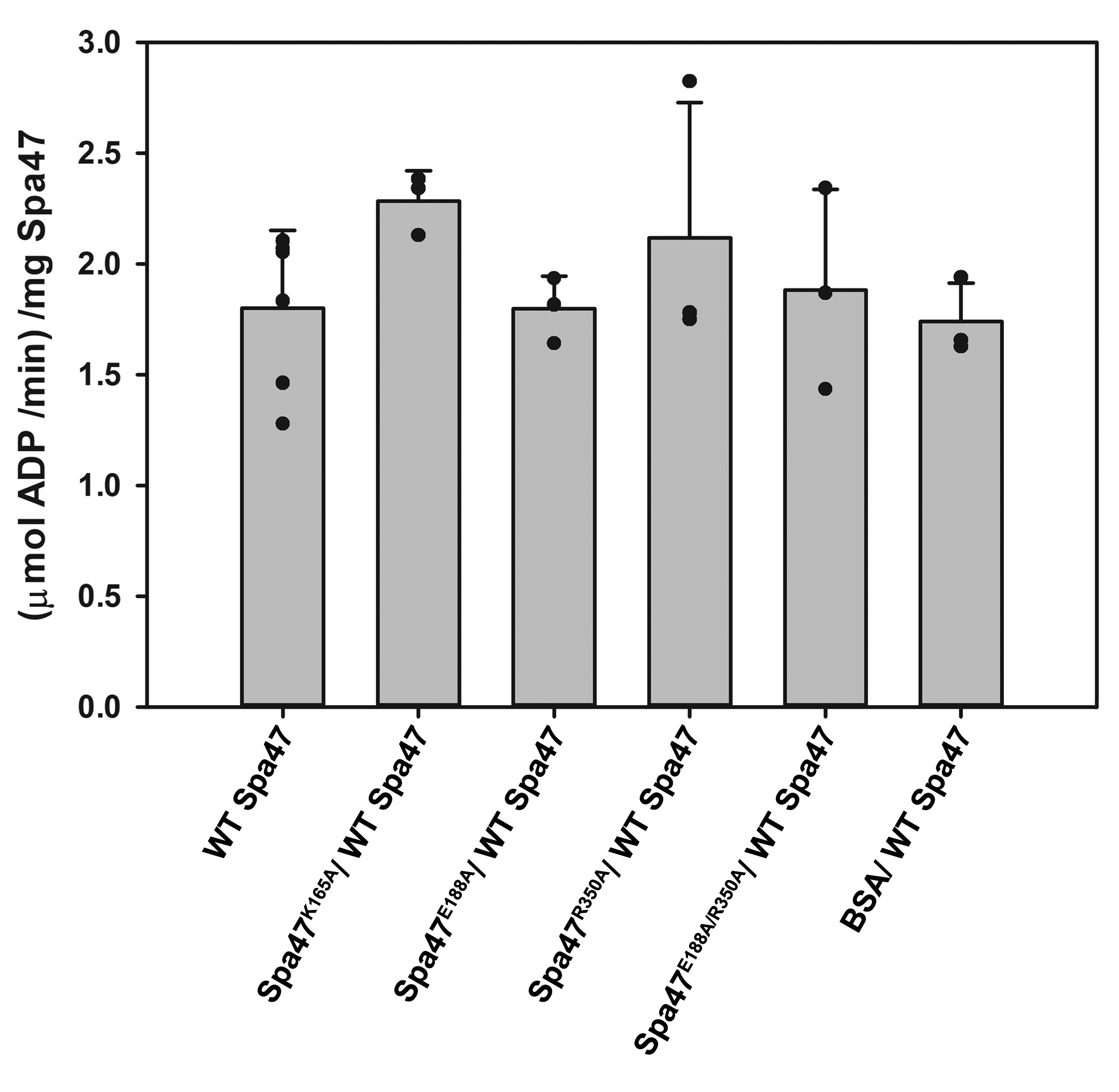

Supplement: S2 Fig — Excess of each ATPase inactive oligomeric Spa47 active site mutant used in this study (1.8 μM) was added to wild-type oligomeric Spa47 (0.45 μM) prior to quantifying ATP hydrolysis by the Spa47 mixture. The presented data represent the mean ± standard deviation of triplicate measurements. The ATPase activity of the wild-type Spa47 oligomer was unaffected by the addition of the ATPase inactive Spa47 mutants (one-way ANOVA, p ≤ 0.05), suggesting that the oligomers are stable and do not undergo protomer exchange in vitro. (TIF) [file pone.0228227.s002.tif]

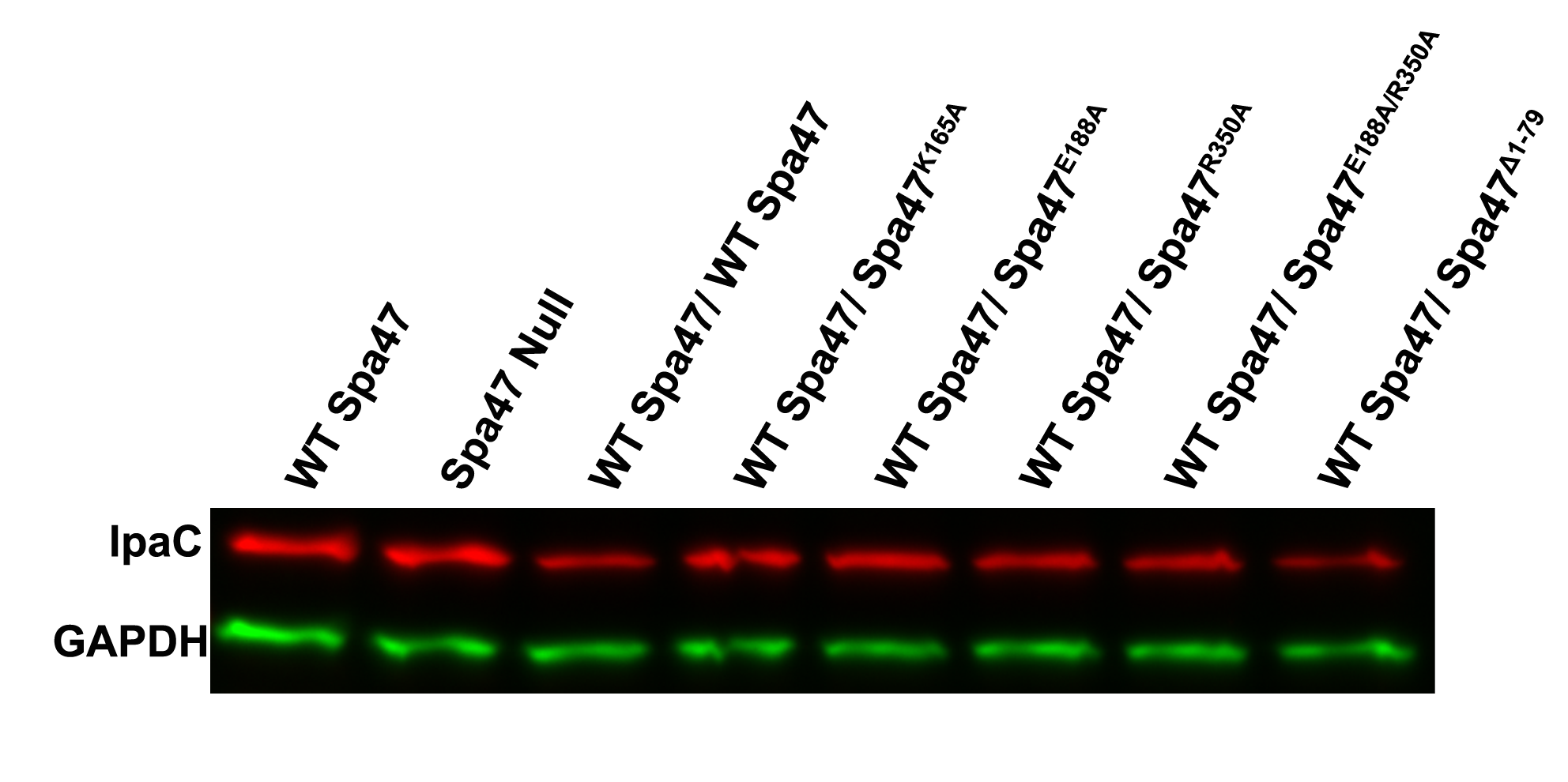

Supplement: S3 Fig — Congo red induction of T3SS effector secretion was performed and the secretion profiles shown in Fig 5. Following isolation of the Shigella culture supernatant, the bacterial cells were lysed and probed via SDS-PAGE/Western blot analysis for cytoplasmic levels of the effector protein IpaC (red) and the cytoplasmic control glyceraldehyde-3-phosphate dehydrogenase (GAPDH, green). (TIF) [file pone.0228227.s003.tif]
