# Supplementary material for: Dominant negative effects by inactive Spa47 mutants inhibit T3SS function and Shigella virulence
Source: PLoS One. 2020 Jan 24;15(1):e0228227. doi: 10.1371/journal.pone.0228227 (PMC6980540; doi:10.1371/journal.pone.0228227)
Supplement: S1 Table — aThe ability of each tested Shigella strain to invade cultured host cells was measured by a standard gentamicin protection assay. Invasion results are presented as the percent invasion by the S. flexneri strain expressing wild-type Spa47. Experiments were performed in triplicate and results represent the mean ± standard deviation of three independent biological replicates. (DOCX) [file pone.0228227.s004.docx]

**S1 Table. Cellular invasion phenotype of *Shigella* *flexneri* expressing an engineered N-terminal GFP Spa47 chimera.**

| *S. flexneri* strain | Complementation | Relative Invasion^a^ (% ± SD) |
| --- | --- | --- |
| *spa47* null | Spa47/pWPsf4 | 100 |
| *spa47* null | None | 1 ± 1 |
| *spa47* null | *GFP-spa47*/pWPsf4 | 113 ± 15 |

**Table 1.** ^a^The ability of each tested *Shigella* strain to invade cultured host cells was measured by a standard gentamicin protection assay. Invasion results are presented as the percent invasion by the *S. flexneri* strain expressing wild-type Spa47. Experiments were performed in triplicate and results represent the mean ± standard deviation of three independent biological replicates.
