# Supplementary material for: Dominant negative effects by inactive Spa47 mutants inhibit T3SS function and Shigella virulence
Source: PLoS One. 2020 Jan 24;15(1):e0228227. doi: 10.1371/journal.pone.0228227 (PMC6980540; doi:10.1371/journal.pone.0228227)
Supplement: S2 Table — (DOCX) [file pone.0228227.s005.docx]

| Bacterial Strain | Relevant Genotype | Expression Plasmid | Reference |
| --- | --- | --- | --- |
| *S. flexneri* | *Spa47 null* | None | 1 |
| *S. flexneri* | *Spa47* | pWPsf4 | 2 |
| *S. flexneri* | *Spa47^K165A^* | pWPsf4 | 2 |
| *S. flexneri* | *Spa47^E188A^* | pWPsf4 | 3 |
| *S. flexneri* | *Spa47^R350A^* | pWPsf4 | 3 |
| *S. flexneri* | *Spa47^E188A/R350A^* | pWPsf4 | This study |
| *S. flexneri* | *Spa47^Δ1-79^* | pWPsf4 | 3 |
| *S. flexneri* | *GFP-Spa47* | pWPsf4 | This study |
| *S. flexneri* | *Spa47/Spa47* | Polycistronic pWPsf4 | This study |
| *S. flexneri* | *Spa47/Spa47^K165A^* | Polycistronic pWPsf4 | This study |
| *S. flexneri* | *Spa47/Spa47^E188A^* | Polycistronic pWPsf4 | This study |
| *S. flexneri* | *Spa47/Spa47^R350A^* | Polycistronic pWPsf4 | This study |
| *S. flexneri* | *Spa47/Spa47^E188A/R350A^* | Polycistronic pWPsf4 | This study |
| *S. flexneri* | *Spa47/Spa47^Δ1-79^* | Polycistronic pWPsf4 | This study |
| *S. flexneri* | *Spa47/GFP-Spa47* | Polycistronic pWPsf4 | This study |
| *S. flexneri* | *Spa47/GFP-Spa47^K165A^* | Polycistronic pWPsf4 | This study |
| *S. flexneri* | *Spa47/GFP-Spa47^E188A^* | Polycistronic pWPsf4 | This study |
| *S. flexneri* | *Spa47/GFP-Spa47^R350A^* | Polycistronic pWPsf4 | This study |
| *S. flexneri* | *Spa47/GFP-Spa47^E188A/R350A^* | Polycistronic pWPsf4 | This study |
| *S. flexneri* | *Spa47/GFP-Spa47^Δ1-79^* | Polycistronic pWPsf4 | This study |
| *E. coli* Tuner(DE3) | *WT Spa47* | pTYB21 | 2 |
| *E. coli* Tuner(DE3) | *Spa47^K165A^* | pTYB21 | 2 |
| *E. coli* Tuner(DE3) | *Spa47^E188A^* | pTYB21 | 3 |
| *E. coli* Tuner(DE3) | *Spa47^R350A^* | pTYB21 | 3 |
| *E. coli* Tuner(DE3) | *Spa47^E188A/R350A^* | pTYB21 | This study |
| *E. coli* Tuner(DE3) | *Spa47^Δ1-79^* | pTYB21 | 3 |

**Supplementary Table S2.** Strains and plasmids used in this study.

**References:**

**1.** Jouihri N, Sory MP, Page AL, Gounon P, Parsot C, Allaoui A. MxiK and MxiN interact with the Spa47 ATPase and are required for transit of the needle components MxiH and MxiI, but not of Ipa proteins, through the type III secretion apparatus of Shigella flexneri. Mol Microbiol. 2003;49(3):755-67. 10.1046/j.1365-2958.2003.03590.x. PMID: 12864857.

**2.** Burgess JL, Jones HB, Kumar P, Toth RT, Middaugh CR, Antony E, Dickenson NE. Spa47 is an oligomerization-activated type three secretion system (T3SS) ATPase from Shigella flexneri. Protein Science: A Publication of the Protein Society. 2016;25(5):1037-48. 10.1002/pro.2917. PMID: 26947936.

**3.** Burgess JL, Burgess RA, Morales Y, Bouvang JM, Johnson SJ, Dickenson NE. Structural and Biochemical Characterization of Spa47 Provides Mechanistic Insight into Type III Secretion System ATPase Activation and Shigella Virulence Regulation. J Biol Chem. 2016;291(50):25837-52. 10.1074/jbc.M116.755256. PMID: 27770024.
